# The Application Effect of Craniotomy through Transsylvian Rolandic Point-Insular Approach on Hypertensive Intracerebral Hemorrhage in Posterior Basal Ganglia

**DOI:** 10.1155/2023/2266691

**Published:** 2023-11-29

**Authors:** Guobing Wang, Xin Chen, Linghu Meng, Ying Liu, Yongjian Dai, Wenxin Wang

**Affiliations:** ^1^Department of Neurosurgery, Renmin Hospital, Hubei University of Medicine, Shiyan, Hubei 442000, China; ^2^Department of Gynecology and Obstetrics, Renmin Hospital, Hubei University of Medicine, Shiyan, Hubei 442000, China

## Abstract

**Objective:**

To evaluate the hematoma clearance and safety of small bone window craniotomy through the lateral fissure Rolandic point-insular lobe approach for patients with hypertensive intracerebral hemorrhage (HICH) in posterior basal ganglia.

**Methods:**

This retrospective study enrolled a total of 86 patients with HICH in the posterior basal ganglia region who underwent surgery between January 2020 and December 2021. These patients were divided into two groups: the conventional group and the study group. The intraoperative information, postoperative hematoma clearance rate, increasing rate of cerebral edema and rebleeding occurrence rate, postoperative complication rate, and prognoses were compared between the two groups. Additionally, we observed and compared the rate of postoperative cerebral hematoma increase, as well as the neurological function and activities of daily living (ADL) at admission, 3 months, and 6 months after surgery in both groups. Univariate and multivariate logistic regression analyses were performed to explore factors affecting the prognosis of patients with HICH in the posterior basal ganglia region after small bone window craniotomy through the lateral fissure Rolandic point-insular lobe approach.

**Results:**

The study group exhibited significantly shorter automatic eye-opening times and hospital stays compared to the conventional group (*P* < 0.05). Furthermore, the study group demonstrated better hematoma clearance rates, lower rates of cerebral hematoma at postoperative 48 h and 72 h, and lower rates of rebleeding compared to the conventional group (*P* < 0.05). At 3 and 6 months postsurgery, the study group exhibited a significantly greater improvement in neurological function and ADL compared to the conventional group (*P* < 0.05). Additionally, the incidence of postoperative complications in the study group was lower than that in the conventional group (*P* < 0.05). Furthermore, the prognosis of the study group was significantly better than that of the conventional group at the 6-month follow-up (*P* < 0.05).

**Conclusion:**

A small bone window craniotomy via transsylvian Rolandic point-insular approach has been shown to improve the hematoma clearance rate in patients with HICH in the posterior basal ganglia region while also reducing the incidence of complications. This approach is highly safe and feasible for implementation in clinical practice.

## 1. Introduction

Hypertensive intracerebral hemorrhage (HICH) is a severe and acute neurosurgical disease with a high disability rate and fatality rate, accounting for 21% to 48% of all stroke patients [1]. It is often attributed to rupture of intracranial aneurysms [2]. Common clinical findings include focal neurologic deficits at onset, gradually progressive deterioration, and the presence of headache, vomiting, and a depressed level of consciousness. Long-term survivors often suffer from permanent deficits, with up to 75% with significant disability, and only 12% to 39% of the survivors have favorable neurological functions recovered [3]. Basal ganglia hemorrhage refers to bleeding from blood vessels in an area of the brain responsible for body movements, sensation, speech, and personality. Basal ganglia hemorrhage is one of the most destructive types of HICH and is very common clinically, accounting for about 70% of HICH [4]. The mechanism of brain injury caused by HICH is brain tissue compressed by hemorrhage and brain tissue edema [5].

The management of acute intracerebral hemorrhage involves prehospital care, airway protection, blood pressure control, hemostatic treatment, seizure and antiepileptic treatment, blood glucose and temperature control, and surgical treatment [6]. However, the role of surgery for most patients with spontaneous intracerebral hemorrhage (ICH) remains controversial, as highlighted by the Guidelines for the Management of Spontaneous Intracerebral Hemorrhage formulated by the American Heart Association/American Stroke Association (AHA/ASA) in 2015.

The theoretical rationale for hematoma evacuation revolves around the concepts of preventing herniation, reducing ICP, and decreasing the pathophysiological impact of the hematoma on surrounding tissue by decreasing the mass effect or the cellular toxicity of blood products. Despite randomized trials comparing surgery to conservative management failing to demonstrate a clear advantage for surgical intervention, surgical treatment is preferred in cases where patients exhibit a relatively large bleeding volume (>30 mL) and a high risk of persistent intracranial hemorrhage. There are still no clear diagnosis and treatment guidelines for basal ganglia intracerebral hemorrhage. Surgery is an essential approach to treating HICH, as it can effectively remove the cerebral hematoma and reduce intracranial pressure. Along with the advancements in medical technology, neural endoscopic technology, the use of minimally invasive surgical techniques (MIS), and small bone window or microbone window hematoma removal surgery have been widespread in the treatment of HICH. Decompressive craniotomy has been found to be a feasible and safe procedure. It may result in better outcomes for a subset of patients with supratentorial ICH as compared to traditional craniotomy treatment [7]. The development of less invasive techniques might allow hematoma evacuation with less damage to viable brain tissue and reduce the rate of secondary complications compared to traditional craniotomy [8]. Another potential advantage of MIS over conventional surgery is faster access to the hematoma, leading to reduced surgical and anesthesia duration, especially in patients with clinical deterioration and elevated increased intracranial pressure (ICP) [9]. However, there are certain differences between different methods of minimally invasive surgeries [10, 11]. To date, no standard surgical procedure has been proven effective for patients with HICH, particularly those with deep hematomas. The small bone window craniotomy via transtemporal cortex approach is the conventional small bone window method for creating a small bone window. Its safety and efficacy have been well established. On the other hand, the small bone window craniotomy via the transsylvian Rolandic point-insular approach is a relatively innovative approach. To investigate the hematoma clearance rate and the safety of the small bone window craniotomy via Rolandic point-insular approach, this study makes a comparison between the two small bone window craniotomies used in patients with hypertensive intracerebral hemorrhage at the posterior basal ganglia region.

## 2. Materials and Methods

This study met the requirements of the Declaration of Helsinki of the World Medical Association. Informed consents were obtained from all patients and (or) their families. This investigation was approved by the Ethics Committee of Renmin Hospital, Hubei University of Medicine.

### 2.1. Subjects

A total of 86 patients with posterior basal ganglia HICH who received treatment in Renmin Hospital, Hubei University of Medicine, from January 2020 to December 2021 were retrospectively selected as the research objects. Patients were divided into the study group and the conventional group, each consisting of 43 cases, based on the inclusion/exclusion criteria and random number table method.

Patients who met the following criteria were included in the study: (1) diagnosis of cerebral hemorrhage according to Guidelines for the Management of Spontaneous Intracerebral Hemorrhage (2015), with patients and (or) their families' willingness, and the subjective indications for surgery therapy 6, without achieving the stability of the ICH; (2) availability of complete imaging material reporting posterior basal ganglia hemorrhage, such as computerized tomography (CT) or magnetic resonance imaging (MRI); (3) the time from the onset to the treatment duration was less than 24 h; (4) hematoma volume ≥ 30 mm; (5) whose lesion was unilateral; and (6) patients without cerebral hernia. Exclusion criteria were as follows: (1) liver and kidney disease (bilirubin ≥ 171 *μ*mol/L, prothrombin activity < 40%, and glomerular filtration rate < 90 mL/min); (2) cerebral arteriovenous malformation; (3) malignant hematonosis; (4) ischemic stroke; (5) hemorrhage caused by intracranial tumor or cerebrovascular malformation; (6) respiratory failure; (7) dilated pupils before surgery; (8) disordered vital signs (any two of the following: (a) body temperature > 37.5°C; (b) rapid pulse ≥ 100 times/min; (c) rapid respiration (>20 times/min) or slow respiration (<12 times/min); (d) systolic blood pressure > 140 mm Hg or diastolic blood pressure > 90 mm Hg; and (e) inconsistent pupil size or disappeared bilateral dilated pupil response to light); and (9) abnormal coagulation function.

### 2.2. Treatment

**Figure 1:**
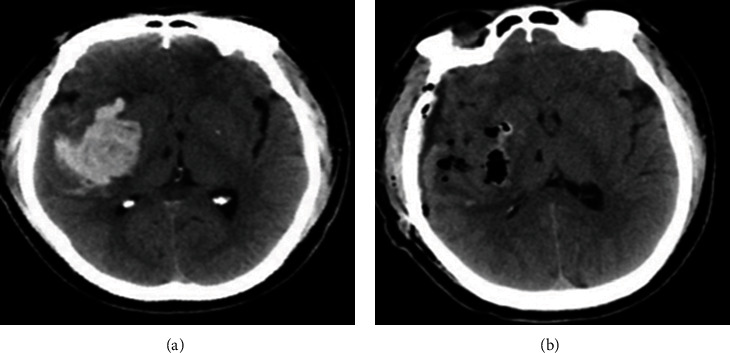
A 54-year-old male patient with hypertensive intracerebral hemorrhage in the posterior basal ganglia region: (a) preoperative CT; (b) postoperative CT.

The conventional group underwent treatment with the small bone window craniotomy via the transtemporal cortex approach, which involved tracheal intubation and general anesthesia. Based on the preoperative head CT examination positioning and the location of the hematoma (Figure 1), the surgical incision was determined. A straight incision of 4-5 cm was made to create a small bone window of 2.5-3.0 cm through milling, and the endocranium was cut through with a cruciform incision. The cortex of either the superior temporal gyrus or middle temporal gyrus was punctured minimally invasively for partial extraction of the hematoma. A fistula measuring 0.5 cm × 1.0 cm was created along the puncture direction. Wet brain cotton was applied to protect the brain tissue, and the fistula was carried out under microscopic observation with due care taken to protect the brain tissue and blood vessels. Once the fistula was deep enough, the hematoma was cautiously extracted, and the hematoma cavity was repeatedly flushed with normal saline to identify any small bleeding points. Electric coagulation was employed to achieve hemostasis until the normal saline became clear. Hemostatic fiber or gelatin sponge was used to fill the hematoma cavity, a drainage tube was inserted, and the brain was closed using routine procedures.

**Figure 2:**
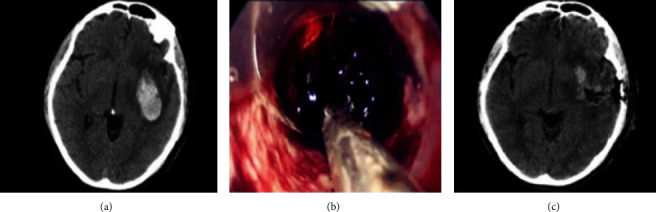
A 54-year-old male patient with hypertensive cerebral hemorrhage in the posterior basal ganglia region: (a) preoperative CT; (b) intraoperative CT; (c) postoperative CT.

Regarding the study group, they were treated with small bone window craniotomy via the transsylvian Rolandic point-insular approach (Figure 2). The anesthesia method was the same as that for the conventional group. Refer to Rhoton [12] for body surface localization of lateral fissure, anterior point of lateral fissure, and lower Rolandic point. Small bone window craniotomy was performed with a straight incision of 6-8 cm slanted backward from the temporal bone in front of the ear. The small bone window craniotomy was performed with the pterion point entering the skull. A 6 cm straight or curved incision in the frontotemporal region was made. A milling cutter was used to mill out a 2.5-3.0 cm small bone window and cut the dura mater in a cross shape to expose the lateral fissure. Under a microscope, a small incision was made through the frontal approach of the lateral fissure, and cerebrospinal fluid was discharged through the lateral fissure arachnoid surgery. The arachnoid membrane adhered to outside the lateral fissure brain tissue was slowly and bluntly separated, and the outer capsule of the blood vessels was dissected. Wet brain cotton was given to protect the brain tissue. The lower Rolandic point was anatomically dissected to expose the middle and rear of the insula and puncture the area without blood vessels on the surface of the insula to determine the hematoma cavity. The bipolar electrocoagulation was used to burn the insula cortex and cut open the insula. After reaching the hematoma cavity, the hematoma cavity was repeatedly rinsed with physiological saline to check for small bleeding points, and electrocoagulation was applied to stop bleeding. If there was old blood, it was removed with a puncture needle after it overflows. A 0.5-1.0 cm incision was made, and separate downwards, and enter the hematoma cavity. During this process, the hematoma cavity was rinsed with cold physiological saline. Under the condition of protecting normal brain tissue as much as possible, low current electrocoagulation should be used to stop bleeding points in the residual cavity, and it was strictly prohibited to forcibly remove a small amount of residual hematoma in the area. After the hematoma was cleared, stop the bleeding, and the dura mater was sutured. Hemostatic fiber or gelatin sponge was then applied to fill the hematoma cavity, followed by the insertion of a drainage tube and routine closure of the brain tissue. In cases where patients exhibit intraoperative brain swelling or cortical collapse that is not evident, a frontotemporal parietal large bone flap decompression procedure (involving an extension of the scalp incision upwards and widening of the bone window) may be performed. Postoperatively, routine treatment measures include the administration of 20% mannitol to reduce intracranial pressure, prophylactic use of anti-infective and neurotrophic drugs, resuscitation therapy, enteral nutrition support, and early initiation of rehabilitation treatment.

### 2.3. Observation Indicators

The primary research indicators in this study included postoperative hematoma clearance rate, rebleeding rate, and the incidence of increased rate of cerebral hematoma at different times after surgery in both groups. Safety indicators included the occurrence of postoperative complications. Univariate and multivariate logistic regression analyses were performed to explore factors affecting the prognosis of patients with HICH in the posterior basal ganglia region after small bone window craniotomy through the lateral fissure Rolandic point-insular lobe approach.

Intraoperative information collected included the duration of surgery, intraoperative blood loss, number of cases requiring frontotemporal parietal decompression, postoperative automatic eye-opening time, decompression time (from the opening of the dura until the end of the operation), and length of hospital stay.

Head CT examination was prior to the surgery and at 6, 24, 48, and 72 h postsurgery, respectively. All CT examinations were performed according to the same standardized protocol, with the preoperative hematoma volume being determined from the CT scan conducted 2 h before the operation. The following calculations were performed to determine the postoperative hematoma clearance rate; hematoma increase rate at 24, 48, and 72 h postsurgery; and rebleeding rate: (1) postoperative hematoma clearance rate = (preoperative hematoma volume − postoperative hematoma volume at 6 h)/preoperative hematoma volume × 100%; (2) postoperative hematoma increase rate at 24 h, 48 h, and 72 h after the surgery = (hematoma volume at 6 h after surgery − hematoma volume at 24 h/48 h/72 h after surgery)/hematoma volume at 6 h after surgery × 100%; and (3) the rebleeding rate was determined by reexamining the head CT scan postoperatively. Specifically, this involved assessing whether the amount of hematoma clearance was less and required a second craniotomy or if the volume of hematoma did not decrease or even increase compared to the preoperative scan. In this study, CT scan results were independently interpreted by two attending physicians who each had over 8 years of work experience. If there was any discrepancy in their interpretations, the chief physician was consulted to make the final determination. The physicians who interpreted the CT scans were blinded to the study grouping of the patients.

*Neurological function*: the National Institutes of Health Stroke Scale (NIHSS) [13] was utilized to assess the degree of neurological impairment in patients upon admission and at 3 and 6 months postsurgery. A higher score on the NIHSS indicates a more severe degree of neurological impairment. Researchers who were assessing patients' neurological impairment were blinded to the grouping of patients.

*Daily living activities*: the Barthel index [14] was used to evaluate patients' activities of daily living, including dressing, toileting, eating, bathing, and other daily activities. This evaluation was conducted upon admission and at 3 and 6 months postsurgery. The total score ranges from 0 to 100, with a higher score indicating better daily living activities.

Postoperative complications include aphasia, visual field defect, epilepsy, intracranial infection, and pulmonary infection.

*Follow-up*: all patients were followed up and observed 6 months after the operation. Glasgow Outcome Scale (GOS) [15] was used to evaluate the clinical prognosis, which was divided into grade 1 (death), grade 2 (persistent vegetative state), grade 3 (severe disability), grade 4 (moderate disability), and grade 5 (good recovery). Patients with GOS scores 4-5 were considered to have a good prognosis.

### 2.4. Statistical Analysis

Statistical analysis was performed using SPSS 21.0. For normally distributed data, descriptive statistics were presented as mean ± standard deviation (mean ± SD), and *t*-tests were used for analysis. For data that did not follow a normal distribution, descriptive statistics were presented as median and quartile M (Q1~Q3), and rank sum tests were used for analysis. Count data were presented as frequency (proportions), unidirectional ordered data were compared using rank sum tests, and disordered data were compared using chi-square tests. A *P* value of less than 0.05 was considered statistically significant.

## 3. Results

### 3.1. Clinical Information

The conventional group consisted of 24 males and 19 females, while the study group consisted of 23 males and 20 females. The average age in the study group was 56.34 ± 12.72 years. In terms of the bleeding site, there were 25 cases of posterior hemorrhage in the right basal ganglia and 18 cases of posterior hemorrhage in the left basal ganglia. The average age in the conventional group was 56.27 ± 12.69 years. Furthermore, the bleeding sites included 24 cases of posterior hemorrhage in the right basal ganglia and 19 cases of posterior hemorrhage in the left basal ganglia. Comparison of general data between the two groups revealed no significant differences, such as the course of hypertension, GCS, and diabetes (*P* > 0.05, Table 1).

### 3.2. The Comparison of Surgery Conditions

There were no significant differences observed between the two groups in terms of operative time, intraoperative blood loss, the number of cases undergoing large decompressive craniectomy, and decompression time (*P* > 0.05). However, the study group had significantly shorter postoperative automatic eye-opening time and hospitalization time compared to the conventional group (*P* < 0.001, respectively), as outlined in Table 2.

### 3.3. The Comparison of Hematoma Clearance Rate, Increasing Rate of Cerebral Hematoma, and Rebleeding Rate

No significant difference was observed between the two groups in terms of the increasing rate of cerebral hematoma at 24 h postsurgery (*P* > 0.05). However, the study group exhibited a significantly lower postoperative hematoma clearance rate, increasing rate of cerebral hematoma at 48 and 72 h after surgery, and rebleeding rate compared to the conventional group (*P* < 0.05, respectively), as presented in Table 3.

### 3.4. The Comparison of Neurological Function and Ability of Daily Living

Upon admission, there was no significant difference observed in scores of neurological function and activities of daily living (ADL) between the two groups (*P* > 0.05, respectively). However, at three and six months after surgery, the neurological function score of the study group was lower than that of the conventional group, with a significant difference (*P* < 0.001, respectively), as outlined in Table 4. Conversely, at three and six months after surgery, the score of ADL in the study group was higher than that in the conventional group (*P* < 0.001, respectively), as presented in Table 4.

### 3.5. The Postoperative Complication and Follow-Up

The study group exhibited a lower incidence of postoperative complications compared to the conventional group (*P* = 0.021), as demonstrated in Table 5. Additionally, at six months after surgery, the prognosis of the study group was significantly better than that of the conventional group, with a significant difference (*P* = 0.042), as illustrated in Table 6.

### 3.6. Univariate Analysis of Factors Affecting the Prognosis of Patients with HICH in the Posterior Basal Ganglia Region after Craniotomy through the Lateral Fissure Rolandic Point-Insular Lobe Approach

Univariate analysis showed that the factors affecting the prognosis of patients with HICH in the posterior basal ganglia region after the craniotomy through the lateral fissure Rolandic point-insular lobe approach included age, admission GCS score, length of hospital stay, neurological function at admission, and activities of daily living. The differences were statistically significant (*P* < 0.05), as shown in Table 7.

### 3.7. Multivariate Analysis of Factors Affecting the Prognosis of Patients with HICH in the Posterior Basal Ganglia Region after Craniotomy through the Lateral Fissure Rolandic Point-Insular Lobe Approach

The prognosis of patients with HICH in the posterior basal ganglia region by using the Roland point island approach craniotomy through the lateral fissure was determined as the dependent variable, and the NIHSS score, age, GCS score at admission, surgical time, neurological function at admission, and activity of daily living were used as the independent variables. The prognosis of patients with HICH in the posterior basal ganglia region after the Roland point island approach craniotomy through the lateral fissure was analyzed by multiple logistic regression analysis. There was a statistically significant difference in age, GCS score at admission, surgical time, and neurological function at admission (*P* < 0.05), as shown in Table 8.

## 4. Discussion

Hypertensive intracerebral hemorrhage frequently occurs in the basal ganglia region, which can cause neuron damage through intracerebral hemorrhage injury, occupying effects, and apoptosis. Neurological impairment can manifest within 3 h of hemorrhage, and secondary brain injury can exacerbate neuronal injury, potentially resulting in mortality [16]. Hence, timely removal of hematoma is an effective measure to reduce sustained brain tissue damage and inhibit neurological deterioration in patients with HICH. The role of surgery for most patients with spontaneous ICH remains controversial, according to the Guidelines for the Management of Spontaneous intracerebral hemorrhage formulated by the American Heart Association/American Stroke Association (AHA/ASA) in 2015 [17]. Randomized trials comparing surgical intervention to conservative therapy have not demonstrated a clear benefit of surgery. However, surgical treatment is preferred when individuals have a significant bleeding volume (>30 mL) and the risk of a persistent cerebral hemorrhage. Hematoma removal via a small bone window is a frequently employed surgical technique. It exhibits similar effectiveness to conventional large bone flap craniotomy in hematoma removal while also reducing operation duration, minimizing strain and damage to normal brain tissue, and lowering the incidence of postoperative complications [18]. This study compares two small bone window craniotomy techniques: the conventional method via the transtemporal cortex approach and a relatively novel method via the transsylvian Rolandic point-insular approach. Currently, the temporal cortex approach is frequently used in clinical practice. However, some studies suggest that cutting through the superior or middle temporal gyrus can result in inadvertent damage to normal brain tissue. Furthermore, fistulation during the operation may lead to local brain tissue ischemia [19]. The transsylvian approach is the latest research hotspot. Many studies demonstrated that the Sylvian fissure was the most prominent anatomic structure on the lateral surface of the human brain. This approach is believed to prevent resection of healthy brain tissue, enable early identification of critical middle cerebral artery branches, and facilitate identification of lenticulostriate perforators [20]. However, some studies believed that the anatomical point of the approach was located in the anterior point of the lateral fissure, with an extensive range and a long path to the posterior part of the basal ganglia, which may cause difficult control of the bleeding point and low complete clearance rate of the hematoma [21]. The inferior Rolandic point is situated along the Sylvian fissure, approximately 2.0 to 2.5 cm posterior to the anterior Sylvian point [22]. From an anatomical perspective, this method may be more appropriate for managing hypertensive intracerebral hemorrhage (HICH) in the posterior basal ganglia area. In this study, we found that performing small bone window craniotomy via the transsylvian Rolandic point-insular approach can enhance the hematoma clearance rate for patients with HICH in the posterior basal ganglia region, while also reducing the occurrence of complications. Furthermore, this approach demonstrated a high level of safety.

Xiubing and Peijun believed that microsurgery via the lateral fissure-insula approach can shorten the postoperative automatic eye-opening time of HICH in the basal ganglia region, and the hematoma clearance rate is high [23]. Yao et al. have highlighted that the lateral fissure lobus insular approach is an effective surgical method for treating hypertensive intracranial hemorrhage in the basal ganglia region, resulting in good prognoses, high levels of safety, and improved living ability of patients [24]. Wang et al.'s study elaborated that transsylvian-transinsular approach for evacuation of intracerebral hematoma demonstrated limited complications, shorter length of hospital stay, and improved long-term efficacy and prognosis [25]. The findings of this study revealed that the study group had a higher postoperative hematoma clearance rate and a lower postoperative rebleeding rate at 48 h and 72 h compared to the conventional group. Moreover, patients in the study group had a shorter postoperative automatic eye-opening time and hospital stay. This may refer to the Rolandic point, which can be separated from the Sylvian fissure either anteriorly or posteriorly, depending on the specific location of the hematoma. This separation can facilitate the adjustment of the direction of the insula incision and create a larger operating space, which is beneficial for removing the hematoma. Furthermore, it is beneficial to open the Sylvian fissure before removing the hematoma and draining cerebrospinal fluid to reduce intracranial pressure and minimize brain tissue damage significantly. Creating a physiological gap during the procedure can help avoid damage to functional areas of the brain, such as those responsible for movement and language, as well as surrounding blood vessels. Ultimately, this can significantly decrease the incidence of cerebral edema and rebleeding. In this operation, the Rolandic point may have separated from the lateral fissure, allowing for separation either anteriorly or posteriorly based on the location of the hematoma. This facilitated the adjustment of the insular lobotomy direction and created a larger operating space, which aided in hematoma removal. Additionally, opening the lateral fissure pool and draining cerebrospinal fluid functional areas such as movement and language were minimized, as was direct damage to surrounding blood before clearing the hematoma significantly reduced intracranial pressure and minimized compression and traction injury to brain tissues. Performing surgery through normal brain tissue spaces causes damage to fund vessels. As a result, the incidence of brain edema and rebleeding was significantly reduced.

Yingshi pointed out that utilizing the transsylvian Rolandic point-insular approach to treat HICH in the posterior basal ganglia offers several advantages, including reducing complications, effectively removing hematoma, and improving neurological function and prognosis [26]. Two commonly used and reliable measures to assess daily living ability and function are the daily living ability scale and the Barthel index. The results of this study showed that the neurological function score of the study group was lower than that of the conventional group at 3 and 6 months after surgery, suggesting that craniotomy through the transsylvian Rolandic point-insular approach can improve the neurological function of patients with HICH in the posterior basal ganglia region. The results of this study showed that 6 months after surgery, the prognosis of the study group was significantly better than that of the conventional group, suggesting that craniotomy through the Rolandic point-insular approach can effectively improve the prognosis of patients with HICH in the posterior basal ganglia region. The Rolandic point is located at the projection point of the lower part of the central sulcus at the lateral fissure, which is 1.6-2.6 cm from the anterior point of the lateral fissure. The deep part is the medial lateral part of the insula, the posterior part of the lenticular nucleus, the posterior branch of the internal capsule, and the thalamus. On the medial side of this area is the insular long gyrus, which has few superficial vessels and can be easily separated. The lower part of the central posterior gyrus is also considered a relatively nonfunctional area. By opening the lateral fissure through the lower Rolandic point, direct access to the insular cortex can be achieved without causing damage to the cortex itself. This approach also provides a concise path to the basal ganglia region, which is more conducive to the postoperative recovery of nerve function.

During craniotomy utilizing the Rolandic point-insular approach, the following considerations should be kept in mind: (1) due to the small size of the bone window and intracranial hypertension, there is a risk of brain tissue being squeezed out of the window. Therefore, it is recommended to first release cerebrospinal fluid by selecting a side arachnoid fissure. If the Rolandic point location is difficult to dissect, water could be used to aid in separating the Sylvian fissure. (2) While separating Sylvian fissure, it is recommended to fill a small gelatin sponge to ensure arachnoid tension and facilitate further separation of side fissure.

The study by Zhang et al. [27] pointed out that the admission GCS score was an influencing factor for patients with hypertensive basal ganglia hemorrhage. Deng [28] showed that admission GCS score and age were influencing factors for the prognosis of patients with hypertensive intracerebral hemorrhage in the basal ganglia region after surgery. The results of this study demonstrated that the prognostic factors for patients with HICH in the posterior basal ganglia region after the Rolandic point-insular approach craniotomy through the lateral fissure included age, admission GCS score, surgical time, admission neurological function, and activity of daily living (*P* < 0.05). Multivariate logistic regression analysis was used to investigate the prognostic factors of patients with hypertensive cerebral hemorrhage in the posterior basal ganglia region after transabdominal fissure Roland point island approach craniotomy. The results showed that the prognosis of patients with hypertensive cerebral hemorrhage in the posterior basal ganglia region after transabdominal fissure Roland point island approach craniotomy was statistically significant (*P* < 0.05), including age, surgical time, GCS score at admission, and neurological function at admission. The reasons were as follows: (1) the older the patient was, the lower the organ function of the body, and the occurrence of basic diseases such as diabetes will also increase, and the brain tissue would shrink to varying degrees, reducing the self-repair function. In addition, age decreased body function, and patients' tolerance to stroke and surgical treatment for cerebral hemorrhage decreases, reducing their recovery ability and affecting their prognosis. (2) The GCS score at admission was influenced by intracranial pressure, degree of brain tissue edema, amount of intracranial hematoma, and degree of brain tissue injury. It was commonly used in clinical practice as a scoring system to evaluate the level of consciousness and severity of disability in patients with brain injury and was closely related to the prognosis after brain injury. (3) The longer the surgery time, the longer the patient's surgical site was exposed to the air, increasing the risk of tissue and organ infections and also affecting prognosis. (4) At admission, neurological function was evaluated using the NIHSS scale, which can effectively assess the degree of neurological impairment in patients and has high reliability and validity.

In summary, the small bone window craniotomy via transsylvian Rolandic point-insular surgery can effectively shorten the automatic eye-opening time and postoperative hospital stays of patients with HICH, increase the clearance rate of hematoma, reduce brain hematoma increase rate and rebleeding rate, improve nerve function and daily life ability, and result in fewer postoperative complications with good prognosis and high security.

### 4.1. Limitations

There are still certain limitations to this study. Firstly, the follow-up period was relatively short, and a more prolonged follow-up may offer more comprehensive understandings of the effect of the two surgical methods on long-term recovery. Secondly, the sample size of this study is relatively small, and a larger sample size would provide more robust statistical power. This is a retrospective study, and there may be some inherent selection bias exited. Thirdly, this study lacks a comparison with other minimally invasive surgery methods, which will be considered in future studies. Fourthly, although neurological follow-up staff and imaging result interpreters were not informed about the patient grouping, the difference in the approach of the two surgical methods made it challenging to achieve the ideal blinding effect and avoid observer bias.

## Figures and Tables

**Table 1:** The general information.

Items	Study group (*n* = 43)	Conventional group (*n* = 43)	*χ* ^2^/*t*	*P*
Gender (male/female, *n*)	23/20	24/19	-0.238	0.812
Bleeding sites (*n*, %)			0.047	0.828
Hemorrhage in the posterior right basal ganglia	24	23		
Hemorrhage in the posterior left basal ganglia	19	20		
Course of hypertension (years)	9.12 ± 2.19	9.09 ± 2.21	0.063	0.947
GCS (scores)	9.18 ± 1.19	9.16 ± 2.21	0.052	0.959
Complications (*n*, %)				
Diabetes	9	7	0.365	0.546
Hypertension	6	4	0.453	0.738
Hyperlipidemia	5	3	0.551	0.713
Coronary heart disease	7	4	0.938	0.333
Smoking	15	13	0.212	0.645
Alcoholism	9	6	0.727	0394
With anticoagulant or antiplatelet therapy	11	10	0.063	0.802

GCS: Glasgow Outcome Scale.

**Table 2:** The comparison of surgery condition.

Items	Study group (*n* = 43)	Conventional group (*n* = 43)	*χ* ^2^/*t*	*P*
The duration time from the onset to the surgery (hours)	4.32 ± 1.01	4.27 ± 1.04	0.226	0.822
Operation duration (hours)	3.44 ± 0.49	3.28 ± 0.51	1.483	0.142
Intraoperative blood loss (mL)	322.79 ± 21.29	328.93 ± 20.97	-1.347	0.182
Cases of large decompressive craniectomy (*n*, %)	1 (2.33%)	3 (6.98%)	1.049	0.306
Eye-opening time (hours)	6.49 ± 0.67	7.71 ± 0.62	-8.764	<0.001^∗^
Decompression time (hours)	1.34 ± 0.21	1.28 ± 0.26	1.177	0.242
Hospital stays (days)	17.38 ± 1.21	18.67 ± 1.23	-4.903	<0.001^∗^

^∗^Compared with the conventional group, *P* < 0.05.

**Table 3:** The comparison of hematoma clearance rate, increased cerebral edema rate, and rebleeding rate.

Items	Study group (*n* = 43)	Conventional group (*n* = 43)	*t*	*P*
Postoperative hematoma clearance rate (%)	94.32 ± 5.29	89.99 ± 5.34	3.777	<0.001^∗^
Increasing rate of cerebral edema (%)				
Postoperative 24 h	1.14 ± 0.21	1.17 ± 0.19	-0.695	0.489
Postoperative 48 h	1.30 ± 0.19	1.61 ± 0.22	-6.993	<0.001^∗^
Postoperative 72 h	1.39 ± 0.23	1.78 ± 0.26	-7.367	<0.001^∗^
Rebleeding rate (*n*, %)	1 (2.33)	8 (18.60)	6.081	0.014^∗^

^∗^Compared with the conventional group, *P* < 0.05.

**Table 4:** The comparison of neurological function and ability of daily living.

	Study group (*n* = 43)	Conventional group (*n* = 43)	*t*	*P*
Neurological function				
At admission	19.21 (9.02, 24.93)	19.12 (9.12, 22.09)	0.402	0.662
3 months after surgery	9.01 (3.02, 13.09)	11.32 (5.83, 15.23)	-5.088	<0.001^∗^
6 months after surgery	3.28 (0.78, 5.98)	4.81 (0.98, 6.93)	-27.688	<0.001^∗^
Ability of daily living				
At admission	32.93 (14.98, 42.09)	32.78 (15.23, 44.09)	0.157	0.876
3 months after surgery	70.87 (67.09, 83.92)	66.01 (63.02, 78.93)	4.388	<0.001^∗^
6 months after surgery	77.82 (68.39, 89.49)	73.29 (65.39, 82.19)	3.769	<0.001^∗^

^∗^Compared with the conventional group, *P* < 0.05.

**Table 5:** The comparison of postoperative complications.

Complications (*n*, %)	Study group (*n* = 43)	Conventional group (*n* = 43)	*χ* ^2^	*P*
Aphasia	0	1 (2.33)		
Visual field defect	1 (2.33)	2 (4.65)		
Epilepsy	0	2 (4.65)		
Intracranial infection	0	1 (2.33)		
Lung infection	1 (2.33)	3 (6.98)		
Total cases	2 (4.65)	9 (20.93)	5.286	0.021^∗^

^∗^Compared with the conventional group, *P* < 0.05.

**Table 6:** The comparison of GOS.

GOS	Study group (*n* = 43)	Conventional group (*n* = 43)	*χ* ^2^	*P*
1 point (*n*, %)	0 (0.00%)	0 (0.00%)		
2 points (*n*, %)	1 (2.32%)	6 (13.95%)		
3 points (*n*, %)	8 (18.60%)	14 (32.56%)		
4 points (*n*, %)	21 (48.84%)	17 (39.53%)		
5 points (*n*, %)	13 (30.23%)	6 (13.95%)		
Good prognosis rate (*n*, %)	34 (79.07%)	23 (53.49%)	8.021	0.042^∗^

Good prognosis: patients with GOS score 4-5 were considered in a good prognosis. GCS: Glasgow Outcome Scale. ^∗^Compared with the conventional group, *P* < 0.05.

**Table 7:** Univariate analysis of factors affecting the prognosis of patients with HICH in the posterior basal ganglia region after craniotomy through the lateral fissure Rolandic point-insular lobe approach.

	Good prognosis (*n* = 34)	Poor prognosis (*n* = 9)	*χ* ^2^/*t*	*P*
Gender (male/female, *n*)	18/16	5/4	14.091	<0.001
Age (years)	55.01 ± 14.87	60.09 ± 11.74	0.947	0.349
Hemorrhage position (*n*)			25.659	<0.001
Right basal ganglia posterior hemorrhage	18	6		
Left basal ganglia posterior hemorrhage	16	3		
Duration of hypertension (years)	8.82 ± 2.05	9.98 ± 2.67	1.416	0.164
GCS score at admission(points)	9.01 ± 2.26	10.87 ± 2.13	2.220	0.032
Complications (*n*)				
Diabetes	8	1	0.663	0.416
Hypertension	5	1	0.077	0.781
Hyperlipidemia	4	1	0.003	0.956
Coronary heart disease	5	2	0.295	0.587
Smoking	12	3	0.012	0.913
Drinking	8	1	0.663	0.416
Anticoagulation and antiplatelet therapy (*n*)	9	2	0.067	0.796
Time from onset to operation (h)	4.32 ± 1.59	4.41 ± 1.63	0.150	0.881
Operation time (h)	3.01 ± 0.78	3.66 ± 0.84	2.189	0.034
Intraoperative bleeding (mL)	320.48 ± 25.49	325.93 ± 23.91	0.577	0.567
Cases of frontotemporoparietal large decompression of bone flap (*n*)	0	1	3.868	0.050
Automatic eye-opening time after surgery (h)	6.38 ± 1.83	7.03 ± 1.73	0.958	0.344
Decompression time (h)	1.31 ± 0.53	1.41 ± 0.47	0.514	0.610
Length of hospital stay (d)	17.28 ± 1.39	18.01 ± 1.46	1.387	0.173
Postoperative hematoma clearance rate (%)	95.12 ± 9.03	91.29 ± 9.67	1.116	0.271
Increased rate of cerebral edema (%)				
Postoperative 24 h	1.11 ± 0.19	1.18 ± 0.29	0.876	0.386
Postoperative 48 h	1.21 ± 0.33	1.29 ± 0.36	0.635	0.529
Postoperative 72 h	1.56 ± 0.37	1.46 ± 0.39	0.713	0.480
Hemorrhage rate (%)	0	1 (11.11)		
Neurological function on admission (points)	19.01 ± 4.11	20.91 ± 4.29	1.223	0.229
Activities of daily living (points)	33.76 ± 4.87	30.98 ± 4.37	1.553	0.128
Occurrence of complications (*n*)				
Aphasia	0	0		
Visual field defect	1	0		
Epilepsy	0	0		
Intracranial infection	0	0		
Pulmonary infection	0	1		
Total occurrence rate	1 (2.94)	1 (11.11)	1.071	0.301

**Table 8:** Multivariate analysis of factors affecting the prognosis of patients with HICH in the posterior basal ganglia region after craniotomy through the lateral fissure Rolandic point-insular lobe approach.

	*β*	SE	Wald *χ*^2^ value	OR (95% CI)	*P* value
Age	0.511	0.249	2.078	1.532 (1.027-2.871)	0.031
GCS score at admission	0.337	0.092	3.609	1.398 (1.138-1.673)	<0.001
Operation time	5.151	1.453	8.082	2.979 (0.906-5.383)	<0.001
Neurological function on admission	0.537	0.126	4.278	1.706 (1.287-2.098)	<0.001
Activities of daily living	0.203	0.243	0.879	1.229 (0.791-1.897)	0.387

## Data Availability

The datasets generated and analyzed during the current study are available from the corresponding author on reasonable request.
